# Blood stream Infections in chronic hemodialysis patients - characteristics and outcomes

**DOI:** 10.1186/s12882-023-03442-5

**Published:** 2024-01-03

**Authors:** Miri Schamroth Pravda, Yasmin Maor, Konstantin Brodsky, Anna Katkov, Relu Cernes, Nili Schamroth Pravda, Milena Tocut, Iris Zohar, Arie Soroksky, Leonid Feldman

**Affiliations:** 1 Department of Intensive care medicine, E. Wolfson Medical Center, 62 Halochamim Street, Holon, 5822012 Israel; 2 Department of Infectious Diseases, E. Wolfson Medical Center, Holon, Israel; 3 Department of Internal medicine D, E. Wolfson Medical Center, Holon, Israel; 4 Department of Nephrology and Hypertension, E. Wolfson Medical Center, Holon, Israel; 5 Department of Cardiology, Rabin Medical Center, Petach Tikvah, Israel; 6 Department of Internal medicine C, E. Wolfson Medical Center, Holon, Israel; 7 Sackler School of Medicine, Tel Aviv University, Tel Aviv, Israel

**Keywords:** Blood stream Infection, Hemodialysis, Mortality

## Abstract

**Introduction:**

Bloodstream Infections (BSI) are a major cause of death and hospitalization among hemodialysis (HD) patients. The rates of BSI among HD patients vary and are influenced by local patient and pathogen characteristics. Modifications in local infection prevention protocols in light of active surveillance of BSI has been shown to improve clinical outcomes. The aim of this study was to further explore factors associated with BSI in a contemporary cohort of HD patients at a public teaching hospital dialysis center in Israel.

**Methods:**

This was a retrospective cohort study of HD patients with a BSI in the years 2014 to 2018. The primary outcome was the occurrence of BSI. Secondary outcomes were to describe the causative pathogens of BSI, and to assess for risk factors for BSI, and mortality.

**Results:**

Included were 251 patients. The mean age was 68.5 ± 13.4 years, 66.9% were male. The mean time from initiation of dialysis was 34.76 ± 40.77 months, interquartile range (IQR) 1-47.5 months and the follow up period of the cohort was 25.17 ± 15.9 months. During the observation period, 44 patients (17.5%) developed 54 BSI events, while 10 of them (3.9% of the whole cohort) developed recurrent BSI events. Gram-negative microorganisms caused 46.3% of all BSI events. 31.4% of these BSI were caused by resistant bacteria. In a multivariate logistic regression analysis, patients receiving dialysis through a central line had a significantly increased risk for BSI adjusted Odds Ratio (aOR) 3.907, p = 0.005, whereas patients’ weight was mildly protective (aOR 0.971, p = 0.024).

**Conclusions:**

We noted an increased prevalence of gram-negative pathogens in the etiology of BSI in HD patients. Based on our findings, additional empirical antibiotics addressing gram negative bacteria have been added to our empirical treatment protocol. Our findings highlight the need to follow local epidemiology for implementing appropriate preventative measures and for tailoring appropriate empiric antibiotic therapy.

## Introduction

Bloodstream infections (BSI) are well recognized as an important cause of morbidity and mortality amongst hemodialysis patients (HD) [1]. Patients on HD are known to have a higher morbidity and mortality rates from BSI compared to the general population [2, 3]. The annual mortality due to sepsis, a severe complication of BSI, in this population is 50–100 times higher than that of the general population [4, 5]. The most commonly reported pathogens isolated from BSI in HD patients are *Staphylococcus aureus*, usually with resistance to methicillin and Coagulase negative staphylococci (CONS) and other gram positive pathogens [6, 7].

Patients on HD are at increased risk for BSI due to a multitude of factors including underlying comorbidities, impaired immunity and the need for vascular access for HD therapy with the ensuing breach of skin barriers and increased exposure to external and skin pathogens [8, 9]. In recent years, several preventive measures were introduced in order to reduce vascular access related BSIs [10]. Despite this, the rate of BSI remains high [8, 11]. Active surveillance of BSI to improve prevention protocols has been associated with reduced BSI rate and improved clinical outcomes [12].

The aim of the present study was to investigate characteristics of BSI in HD patients and to further explore the cause and risk factors of these infections.

## Methods

This is a retrospective study of patients who underwent chronic HD between January 2014 and December 2018 at E. Wolfson Medical Center. The cohort included patients aged ≥ 18 years who were treated with HD for end-stage renal disease. Included patients were treated with HD for at least three months. This was done to avoid including patients with recovered kidney function following a short period of HD and who were not chronically treated with HD. We excluded patients undergoing HD due to acute kidney injury, patients with peritoneal dialysis or patients treated with HD for less than 3 months. The study period was defined from the day of HD initiation until the date of the last follow up. Follow up data was completed until December 2018. During this period, standard infection control practices were employed in the hemodialysis unit to decrease the rate of BSI. These include strict adherence to hand hygiene protocols, screening of patients for colonization with multidrug resistant pathogens (MDR) (methicillin resistant *Staphylococcus aureus*, and carbapenem resistant *enterobacterales*, *Clostridium difficile*) strict contact isolation of patients colonized with MDR pathogens. Central lines were attended to according to infection control recommendations in a sterile manner. Data were retrieved by retrospective review of the patients’ medical electronic records. The sociodemographic details, comorbidities, infection events, hospitalizations and mortality data were summarized from the patients’ files. We also collected data regarding mode of dialysis, and quality of the dialysis. All included patients were treated with chronic ambulatory hemodialysis in a public hospital-based facility. Patient dialysis regimen is usually three times per week for four hours per HD treatment, however each regimen is individualized according to volume-status and electrolyte considerations. We included data regarding the pathogens identified from all blood cultures of patients during the study period. We also collected data regarding resistance to antibiotics.

The primary outcome was the occurrence of BSI. Secondary outcomes were to describe the causative pathogens of BSI, to assess for risk factors for BSI, and mortality, and risk factors for mortality amongst this cohort.

The primary outcome examined was BSI rate and type amongst the cohort. BSI was defined as a laboratory confirmed bloodstream infection according to the CDC (Center for Disease Control and Prevention) criteria [13]. When an access infection was suspected, two sets of blood cultures were taken. Hospital guidelines recommend taking a peripheral blood culture and if a central line is present taking also a blood culture from the central line. Blood was drawn and immediately placed in blood culture bottles and these were transported to the microbiology laboratory where the bottles were placed in designated incubators assessing carbon dioxide emission. Once there was a signal of bacterial growth, a sample was placed in the Vitek system for bacteria identification and for assessment of susceptibility to antibiotics. Some of the tests were performed manually according to CLSI guidelines using antibiotic discs and/or etests. Susceptibility was defined according to CLSI criteria [14]. Patients in this cohort had primary bacteraemia and their infectious process was not secondary to other focus of infection.

The empirical treatment given to patients with bacteraemia related to dialysis during the period of this study was vancomycin. The dialysis catheter, when present, was taken out in cases of repeated positive culture or in patients with septic shock unresponsive to hydration. Once the pathogen was identified and antibiotic susceptibility was known, the empirical treatment was changed to the most narrow-spectrum antibiotic that was appropriate for the pathogen. Treatment duration was according to the pathogen isolated and patients’ clinical condition. At the time the study was performed we did not use antibiotic locks. When appropriate the central catheter was removed. In *Staphylococcus aureus* bacteremia, patients also underwent echocardiography.

The risk factors assessed for risk of BSI included sex, age, marital status, hepatitis C status, hepatitis B status, vascular access for HD, hemoglobin levels, albumin levels, Kt/V, Background of cancer, smoking, hypertension, diabetes, heart failure, ischemic heart disease, stroke, coagulation disorder.

Data on mortality was based on notification of death from the Ministry of Interior Affairs. We also assessed the risk factors for mortality amongst the cohort.

### Statistical analysis

Patients’ characteristics were presented as n (%) for categorical variables, and as mean with standard deviation (SD) or median [interquartile range - IQR] as appropriate. Continuous variables following a normal distribution were compared using Student’s t-test, whereas those not following a normal distribution are presented as median and interquartile range and were compared using the Mann-Whitney U test. Categorical variables are reported as counts and percentages. The valid percentage was reported. The Spearman correlation was used to explore the continuous relationship between various variables and mortality. Bacteremia rate was calculated by dividing the number of infections by the number of patients. We also calculated the rate of infections per 1,000 catheter days. The risk of developing bacteremia was calculated by bivariate, Spearman correlation between two variables.

A multiple logistic regression was performed to assess for risk factors for mortality. We first performed a bivariate analysis between mortality and the candidate variables. Variables associated with mortality were entered into the logistic regression model (backward stepwise conditional), the dependent variable being mortality. P value for entry was 0.05 and for removal 0.10. In a similar manner we also calculated a logistic regression model where the dependent variable was bacteremia.

All tests were conducted at a two-sided alpha level. P < 0.05 was considered statistically significant. All statistical analyses were performed using IBM SPSS Statistics for Windows, Version 28.0 (Armonk, NY: IBM Corp, 2021).

## Results

The study involved 251 chronic hemodialysis patients. The baseline patient characteristics are presented in Table 1.

**Table 1:** Baseline patients’ characteristics

Variables	N (%)
Patients, n	251
Gender (male)	168 (66.9%)
Age, mean (years)	68.5 ± 13.4
Hemodialysis Vintage (months)	34.76 ± 40.77
**Vascular access, %**	
Catheter	126(56.1%)
Fistula	87 (39.0%)
Graft	11 (4.9%)
**Comorbidities, %**	
Diabetes mellitus	119 (51.1%)
Hypertension	192 (82.4%)
Heart Failure	68 (29.3%)
Coronary disease	94 (40.3%)
Stroke	38 (16.4%)
Smoking	56 (24.1%)
Cancer	40 (17.2%)
Connective Tissue Disease	9 (3.9%)
Weight, kg	72.9 ± 16.6
Hemoglobin, g/L	10.5 ± 1.7
Albumin, g/dL	3.56 ± 0.51
Kt/V, mean	1.36 ± 0.28
**Cause of End Stage Renal Disease, %**	
Diabetes	100 (45.5%)
Hypertension	58 (26.4%)
Vasculitis	6 (2.7%)
Autosomal Dominant Polycystic Kidney Disease	6 (2.7%)
Glomerulonephritis	18 (8.2%)
Other Causes	32 (14.5%)

### Patient characteristics

The average age was 68.5 ± 13.4 years and the majority of the cohort were male (66.9%). The mean time from initiation of dialysis at baseline was 34.76 ± 40.77 months, IQR 1-47.5 months and the mean follow up period of the cohort was 25.17 ± 15.9 months. The cause of end-stage renal disease was most frequently due to diabetes (45.5%) followed by hypertension (26.4%) and glomerulonephritis (8.2%). At baseline, central vein catheter (CVC) was the most frequent type of vascular access for HD (56.1%), followed by arterio-venous fistula (AVF) (39.0%) and by arterio-venous graft (AVG) (4.9%).

### Bloodstream Infections

During the observation period, 44 patients (17.5%) developed 54 bacteremia events, while 10 of them (3.9% of the whole cohort) developed recurrent bacteremia events. (Table 2). The rate of BSI was 0.22 events for the entire cohort and 0.35 BSI per 1,000 catheter days. The rate of the first BSI event (omitting recurrent BSI events) was 0.17 events for the entire cohort, 0.28 BSI per 1,000 catheter days. The rate of recurrent bacteremia (more than one episode in 5 years) was 0.036 events for the entire cohort and 0.06 per 1,000 catheter days. Of all 54 bacteremia events, *Klebsiella pneumoniae* was the most frequently isolated pathogen (22.2%), followed by *S. aureus* (18.5%), CONS (14.8%), *Escherichia coli* (14.8%) and *Pseudomonas aeruginosa* (9.26%). Of these events, 31.4% were caused by resistant bacteria (Table 2). Gram-negative microorganisms caused 46.3% of all BSI events.

**Table 2:** Infection events of the cohort

	1st infectious event		2nd infectious event		3rd event
	Frequency	% of cohort	Frequency	% of cohort	Frequency
**Total infections**	44	17.5	9	3.6	1 (0.4%)
Gram positive infections	15	6.0	3	1.2	0
*Staphylococcus aureus*	8	3.2	2	0.8	0
CONS	7	2.8	1	0.4	0
Gram negative infections	29	11.6	6	2.4	1 (0.4%)
*Escherichia coli*	7	2.8	1	0.4	0
*Klebsiella pneumoniae*	10	4	2	0.8	0
*Pseudomonas aeruginosa*	4	1.6	1	0.4	0
Others	8	3.2	2	0.8	1 (0.4%)
**Infections due to resistant pathogens**	12	4.6	5	2.0	0
MRSA	8	3.1	1	0.4	0
ESBL	4	1.6	1	0.4	0
CRE	-	-	1	0.4	

CONS – coagulase negative staphylococci; MRSA -Methillin resistant *Staphylococcus aureus*; ESBL -extended spectrum beta lactamases Enterobacterales; CRE -carbapenem resistant Enterobacterales, Others include *Candida Albicans*, *Proteus* Species, *Pseudemonas*

### Risk factors for BSI

In multivariate logistic regression analysis, (Table 3) the risk of acquiring a BSI was increased in patients receiving dialysis through a central line (OR 3.907, p = 0.005), patients with a history of a cerebrovascular accident (OR 2.253, p = 0.069), and patients’ weight was mildly protective (OR 0.971, p = 0.024).

**Table 3:** Multivariate analysis of risk factors for bacteremia

Variables	OR	95% CI	p
Hemodialysis by a central line	3.907	1.518–10.053	0.005
Prior cerebrovascular accident	2.253	0.938–5.412	0.069
Patient weight	0.971	0.946–0.996	0.024

### Mortality

In this cohort, 78 patients died during the follow up period and 12 patients underwent a renal transplantation. Results of univariate logistic regression analysis showed that factors associated with death were patients’ age (OR 14.57, p < 0.001), Diabetes Mellitus (OR 12.34, p < 0.001) and bacteremia (OR 4.1, p = 0.043). In multivariate logistic regression analysis, (Table 4) the risk of mortality was increased with bacteremia (aOR 2.566, p = 0.035), patients’ age (aOR 1.068, p < 0.001), diabetes (aOR 1.019, p = 0.001) and decreased with Kt/V (aOR 0.208, p = 0.025).

**Table 4:** Multivariate analysis of risk factors for mortality

Variables	OR	95% CI	p
Infection	2.566	1.069–6.158	0.035
Age	1.068	1.035–1.102	< 0.001
Kt/V	0.208	0.052–0.822	0.025
DM	1.019	1.010–1.028	< 0.001
CHF	1.899	0.919–3.925	0.083

## Discussion

The aim of the present study was to investigate characteristics of BSI in HD patients – the incidence rates of infection, susceptible patients’ characteristics, causative pathogens and clinical outcomes. The main finding was a significant incidence of BSI events amongst HD patients with nearly half of these events caused by gram-negative bacteria. Increased age and diabetes were associated with an increased risk for mortality whereas Kt/V was associated with a decreased risk of mortality. In our cohort, 17.5% of patients developed BSI during follow up. This is a lower incidence than that reported by other studies. Rteil et al. reported an incidence rate of 32.7% BSI in their HD patients and Sahil et al. reported an incidence of 22.4% BSI in their HD patients [9]. This lower reported incidence of BSI was probably related by successful implementation evidence-based preventive infection control strategies which were intensified in HD units worldwide in recent years [15], such as strict adherence to hand hygiene protocols, screening and isolation of patients with MDR pathogens and *Clostridium difficile*, and sterile attendance to catheters. Despite these measures and similar to other reports almost a third of the BSI were due to resistant organisms [16, 17]. This is an important finding because resistant pathogens are associated with an increased risk of mortality, which may be due to the pathogenicity of the bacteria itself and/or inadequate initial empiric antibiotic therapy [18]. We observed a high percentage of gram-negative bacteria causing 46.3% of all BSI events in our cohort. The most frequently isolated pathogen was *K. pneumoniae*, found in 18.5%, while *E. coli* and *P. aeruginosa* were responsible for 14.8% and 9.26% BSIs, respectively. There is an increasing body of evidence demonstrating worldwide changes in the pattern of pathogens causing BSI in HD patients [19–21]. The high prevalence of gram-negative bacteremia in our study is unusual as BSI in HD patients are mostly caused by gram-positive bacteria [16]. Gram positive bacteria commonly reside on the colonizing areas around the catheter site insertion commonly used to deliver HD. The colonization leads to biofilm formation and a nidus for virulent bacteria [22, 23]. The increase in gram-negative pathogens is probably multifactorial and could be a result of an increase in preventative measures mainly targeting gram-positive organisms. Current strategies, such as intranasal or dialysis catheter exit site application of mupirocin, have been shown to be very effective in preventing gram-positive bacterial infections, especially *S. aureus* infection [24, 25]. It is also possible that some of the gram-negative infections reported in our study were related to the spread of bacteria within the hospital (nosocomial) and inadequate infection control practices. Unfortunately, preventive strategies for gram-negative BSI are less well defined as the mechanism of acquiring these infections still warrants further study. In a cohort study on gram negative bacteria BSI amongst HD patients, the source of infection was more likely to be urinary or abdominal [20]. However, attempts of selective decontamination of the gastrointestinal tract and use of chlorhexidine washing were not consistently effective [25]. Nevertheless, some treatment strategies for central line associated BSI (CLABSI) proved to be more effective for gram-negative than for gram-positive bacteria. Indeed, the clinical success of an antibiotic lock protocol in eradicating CLABSI without removal of the catheter was as high as 87% for gram-negative infections compared to 40% and 75% for *S. aureus* and *S. epidermidis*, respectively [26, 27]. The finding of increased prevalence of gram-negative bacteria and a high percentage of drug resistant organisms may also have implications in the choice of empirical antibiotic treatment for BSI. The results support adding gram-negative coverage for empiric antibiotic therapy among HD patients with suspected BSI. The decisions regarding empirical antibiotic coverage should be taken according to local epidemiology to prevent over and underuse of antibiotics.

We identified bacteremia, older age and diabetes as independent risk factors for mortality amongst HD patients. These findings are concurrent with other studies that have previously described these risk factors in HD patients with BSI [28–30]. We also identified risk factors for bacteremia. Of particular note a central line catheter. This risk factor is well known from other studies and emphasizes the importance of administrating HD through a fistula with or without a graft [29, 30]. Our findings of an increased risk with patients with a history of a cerebrovascular accident may be attributed to the fact that these patients are less mobile and have associated vasculopathy. There is limited data regarding the association between weight and risk of BSI in HD patients. Our findings that patients’ weight was mildly protective are congruent with other studies showing that lower body mass index has a significant association with adverse outcomes including infection-related death in similar populations such as those with end stage kidney disease and peritoneal dialysis [31, 32]. Our study has several limitations. This is a single center study so our results may not be highly generalizable. This study was not designed to investigate or compare different preventive strategies for CLABSI. However, our findings did change our local empiric antibiotic regimen for HD patients. We only assessed BSI and no other types of infections in HD patients. Patient included had a primary bacteremia. Patients underwent the standard evaluations related to focus of infections. However, some patients may have been included were the primary infection was from a secondary focus of infection. In this study we did not follow the clinical course of patients regarding metastasis of the primary bacteremia to other foci. The baseline vascular access was reported as per initial vascular access for HD treatment and therefore these is a predominance of catheter-based vascular access. However, the vascular access was changed to fistula or graft vascular access in many patients during the duration of HD treatment.

We also did not assess the source of infection, source control measures, or appropriateness of antibiotic therapy. Another limitation is that, due to missing data, we could not adjust for potential confounding factors such as antibiotic regimen, catheter indwelling time, and duration of hemodialysis in the logistic regression analysis. The data for cause of death was not available for many patients and was not included in this study. These limitations may be counterbalanced by several strengths. Due to fully computerized patients’ files in this center, there was no missing data. All results of microbiology tests were performed in a single laboratory highly certificated for reliability and consistency of results. Follow-up period was longer relative to other studies published on this topic [12, 33]. In this cohort there was a high percentage of patients with tunneled catheters and this may have increased the overall rate of infections and in particular the rate of gram negative infections. This may also affect the generality of the results.

In conclusion, this study demonstrated a high percentage of gram-negative bacteria causing 46.3% of all BSI events in our HD patients. This result reflects an increasing prevalence of gram-negative pathogens in the etiology of BSI in HD patients. Our findings highlight the need to identify local epidemiology in order to implement appropriate preventative measures and tailor empiric antibiotic therapy in HD patients.

## Data Availability

On request to corresponding author.
